# Supplementary figures and images for: Spatial patterns and environmental influences of COVID-19 outbreaks, post-Omicron
Source: PLoS One. 2026 Feb 10;21(2):e0342510. doi: 10.1371/journal.pone.0342510 (PMC12890149; doi:10.1371/journal.pone.0342510)

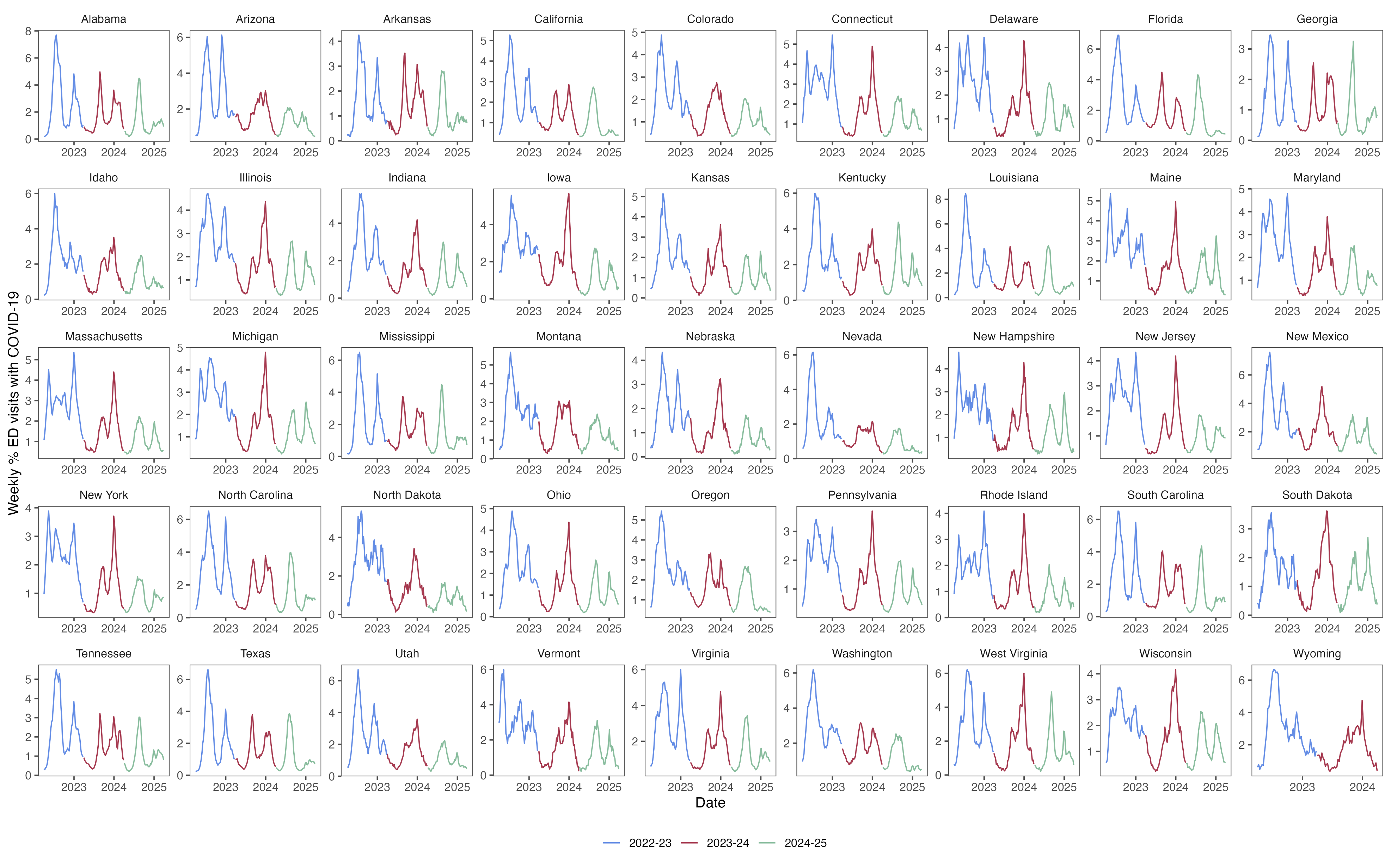

Supplement: S1 Fig — Most states had three years of complete data, while Wyoming stopped reporting in 2025 and thus only used data from April 2022 - March 2024. Each “COVID-19 year” has a different color to indicate the demarcation of the study period. (PNG) [file pone.0342510.s001.png]

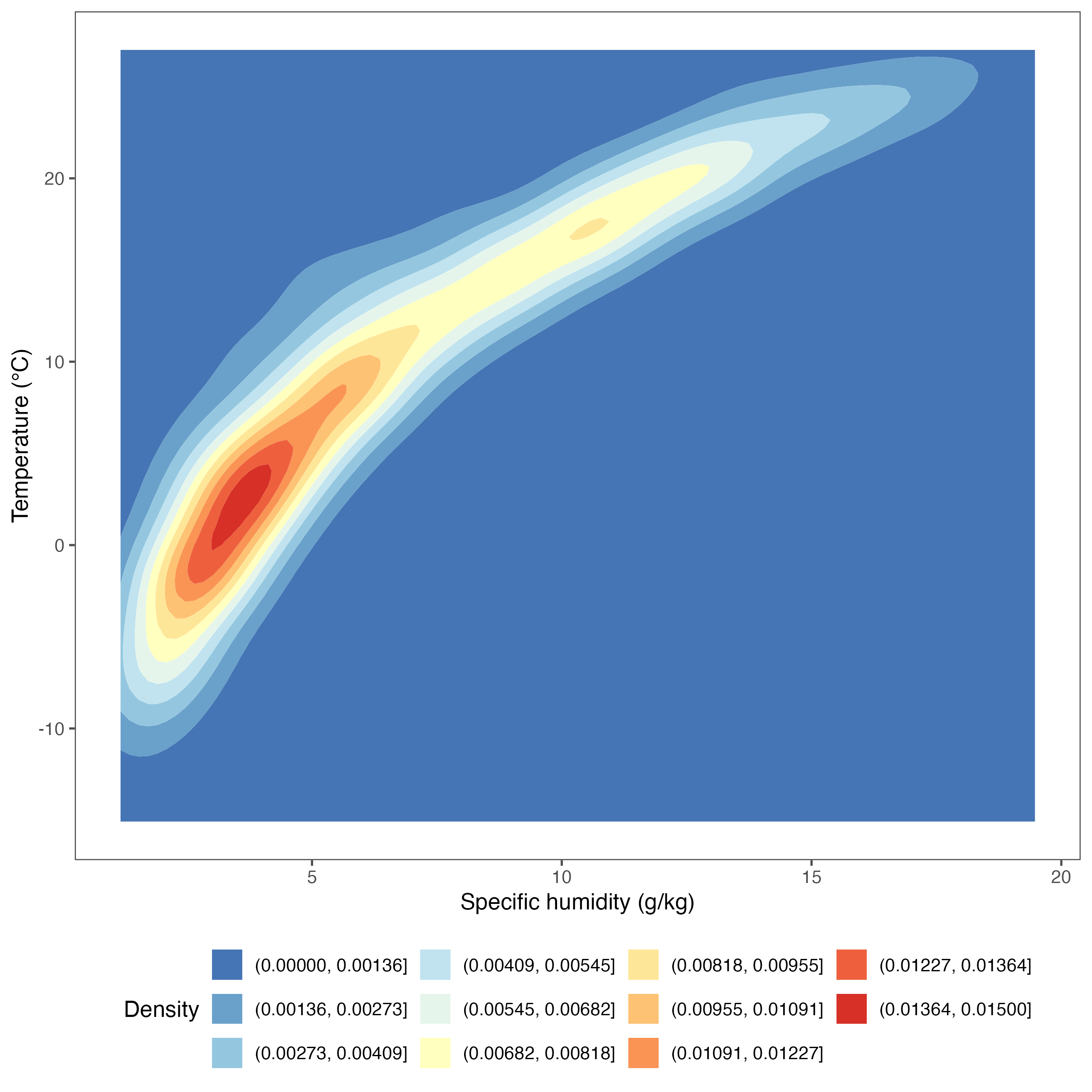

Supplement: S2 Fig — Warmer colors indicate more commonly observed temperature and specific humidity combinations. (PNG) [file pone.0342510.s002.png]

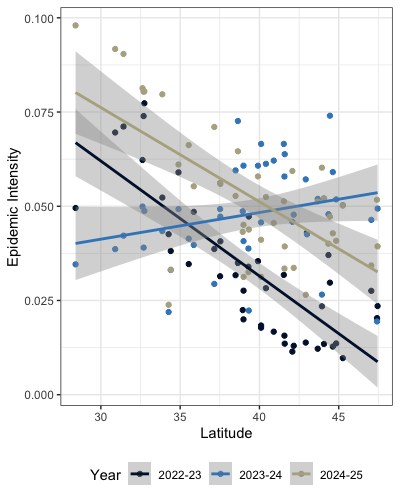

Supplement: S3 Fig — Color indicates year. (PNG) [file pone.0342510.s003.png]

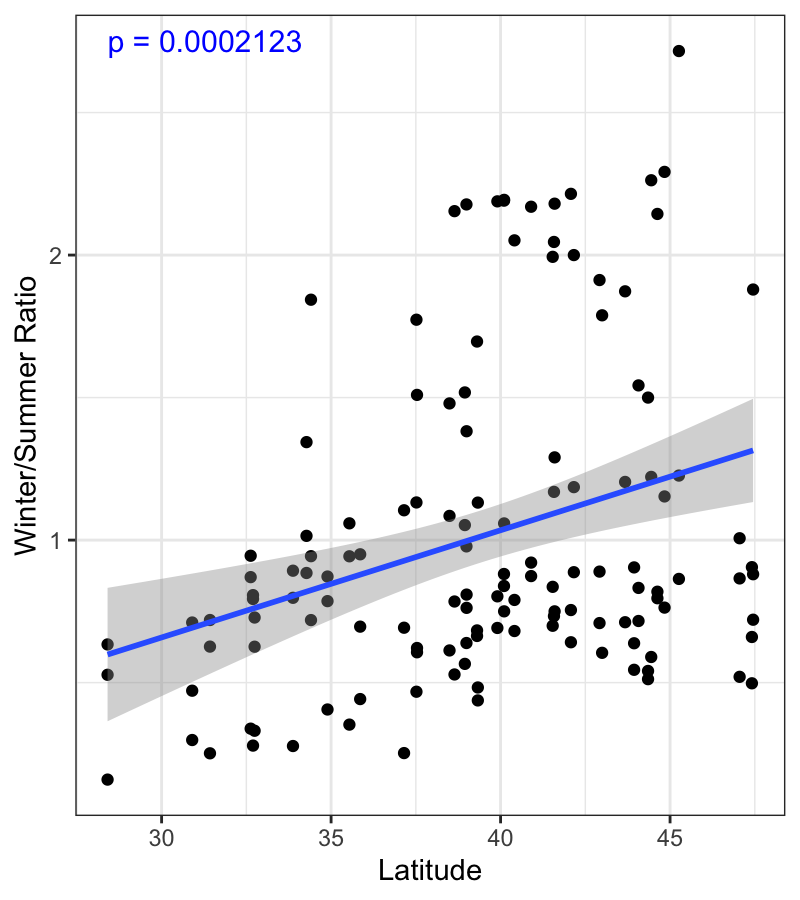

Supplement: S4 Fig — Winter/Summer peak ratio for each state and COVID-year, where larger ratios indicate larger summer peaks relative to winter peaks. (PNG) [file pone.0342510.s004.png]

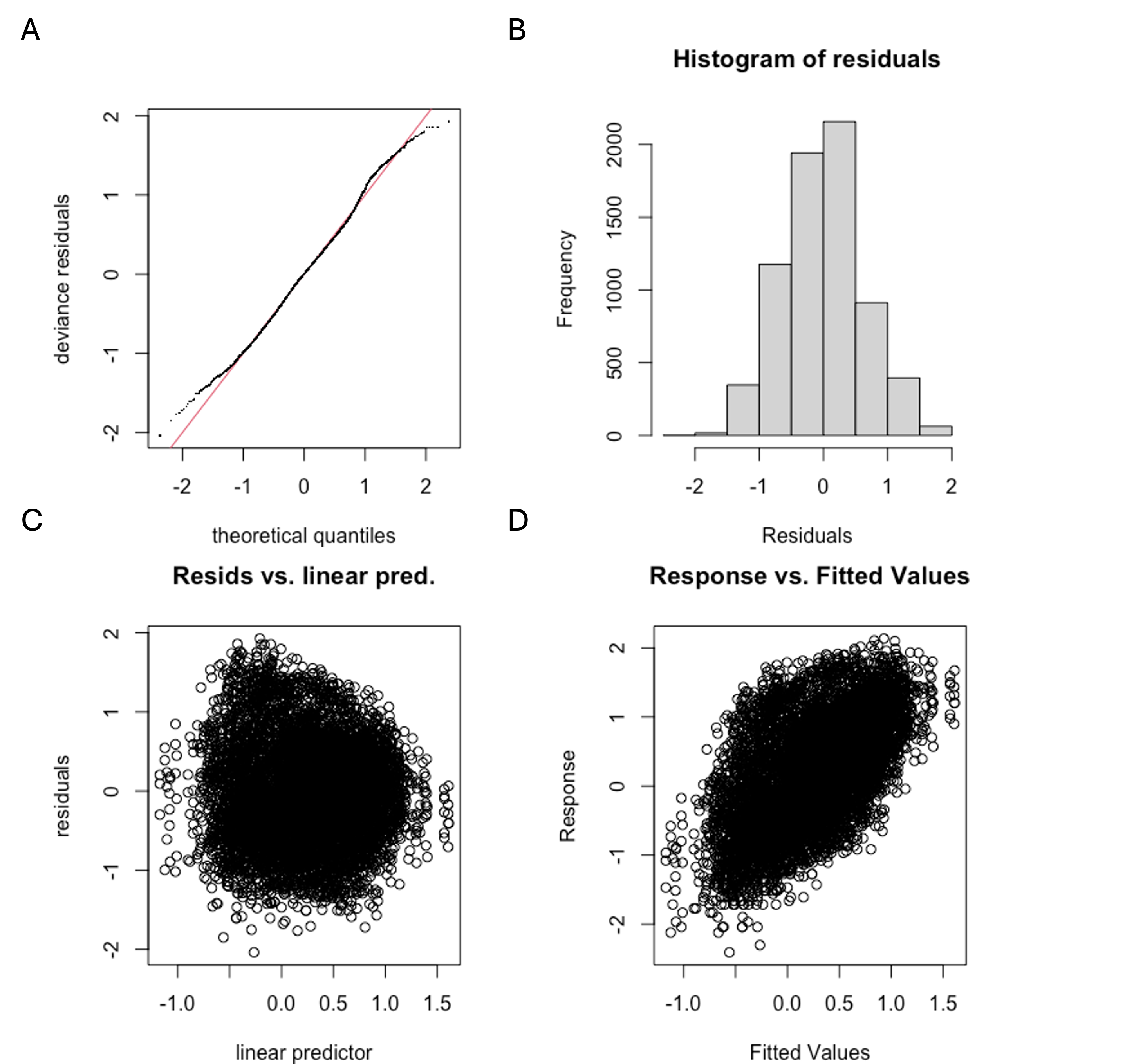

Supplement: S5 Fig — A. Q-Q plot showing residuals largely following the theoretical normal distribution. B. Histogram of residuals, approximately symmetric and centered near zero. C. Residuals versus linear predictor, showing no major nonlinear patterns, with mild heteroskedasticity. D. Observed versus fitted values, indicating that fitted values span a narrower range than the observed log(ED visits). Together, these diagnostics support the adequacy of the model while motivating inclusion of state and cyclic week effects. (PNG) [file pone.0342510.s005.png]

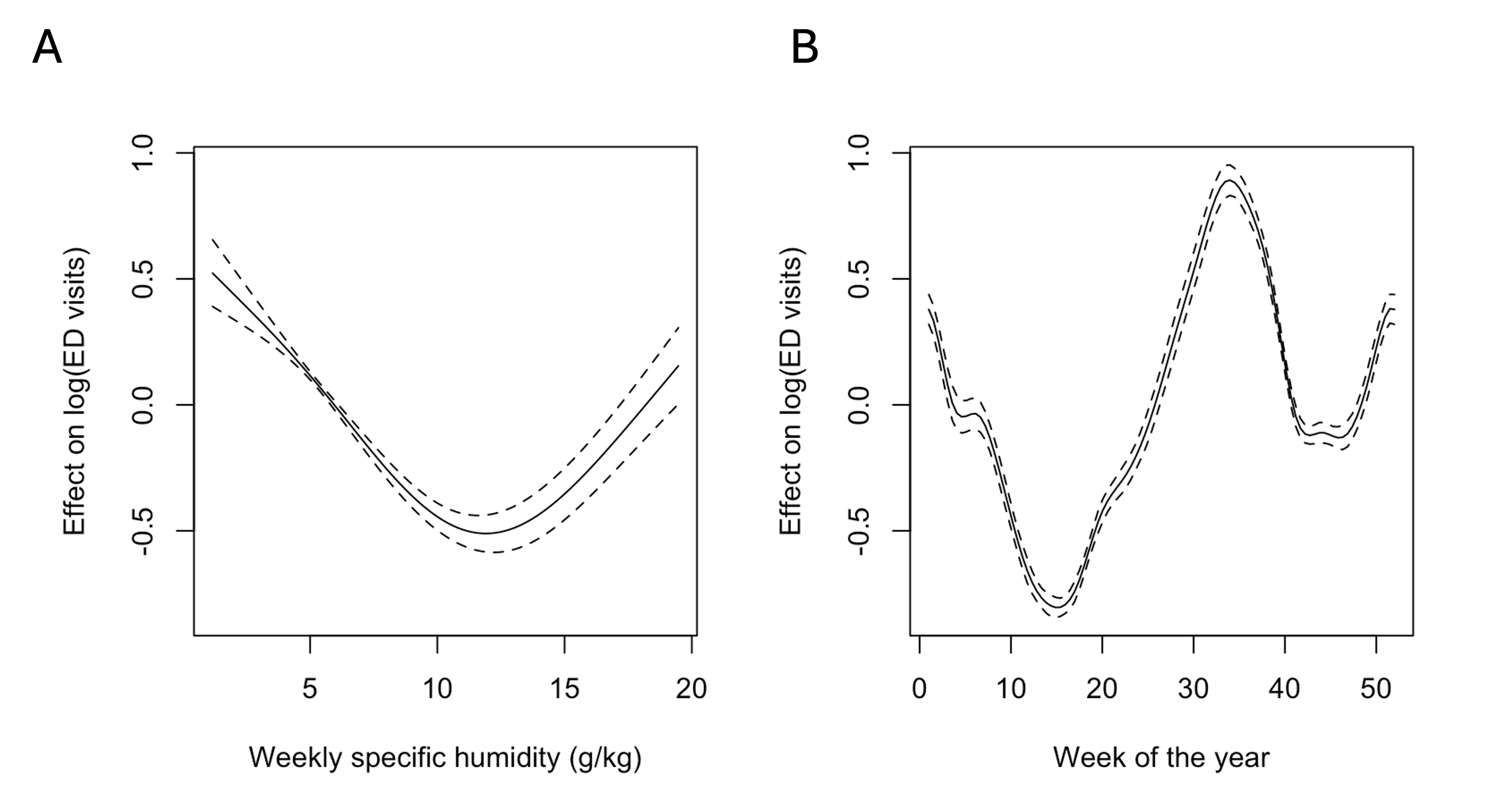

Supplement: S6 Fig — A. Specific humidity shows a nonlinear, U-shaped association with log(ED visits). B. The cyclic spline for week captures within-year seasonal structure. Solid lines represent fitted effects; dashed lines show 95% confidence intervals. (PNG) [file pone.0342510.s006.png]

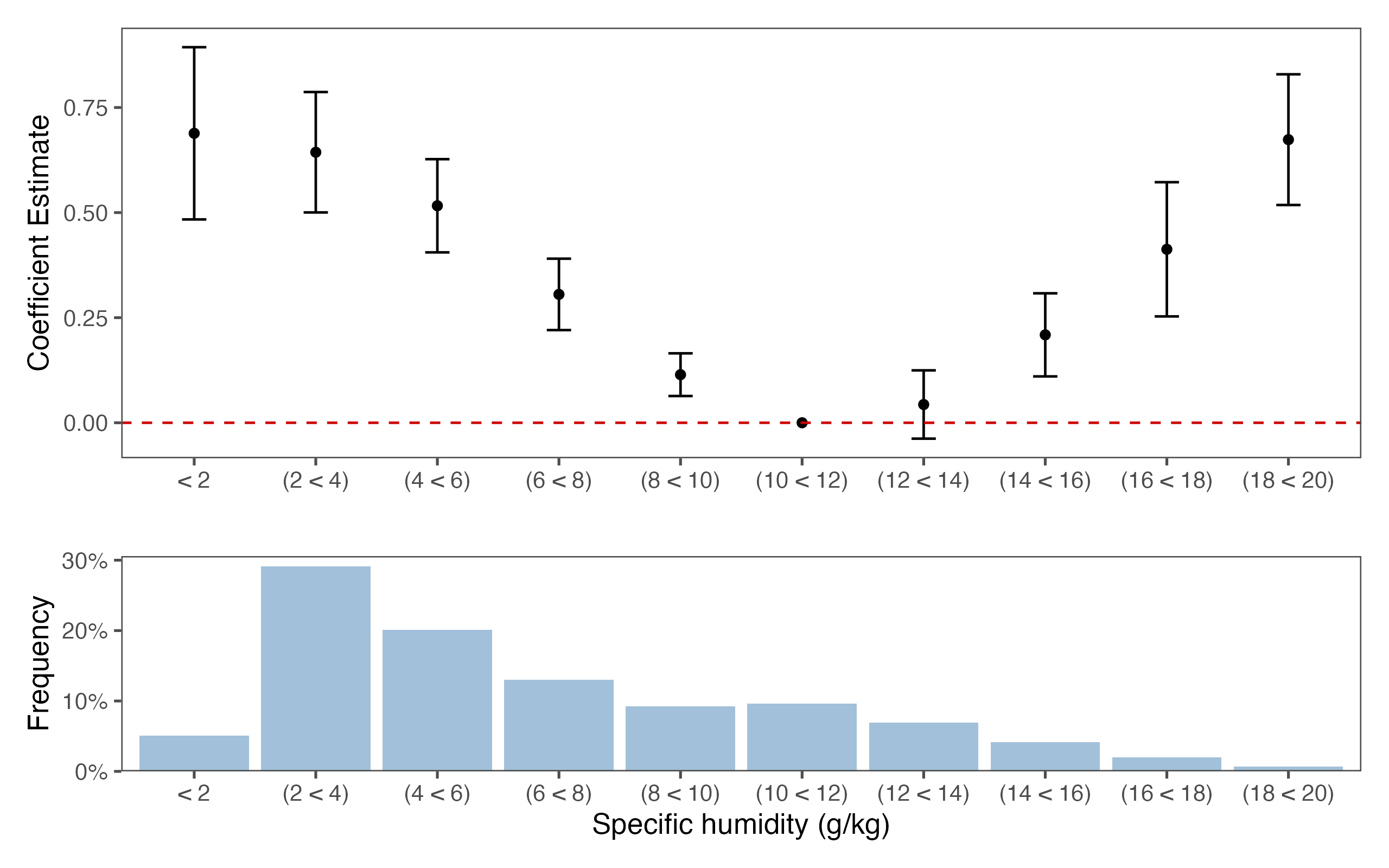

Supplement: S7 Fig — Results from the binned fixed effects model,showing a U-shaped relationship between specific humidity and logged ED visits. (PNG) [file pone.0342510.s007.png]

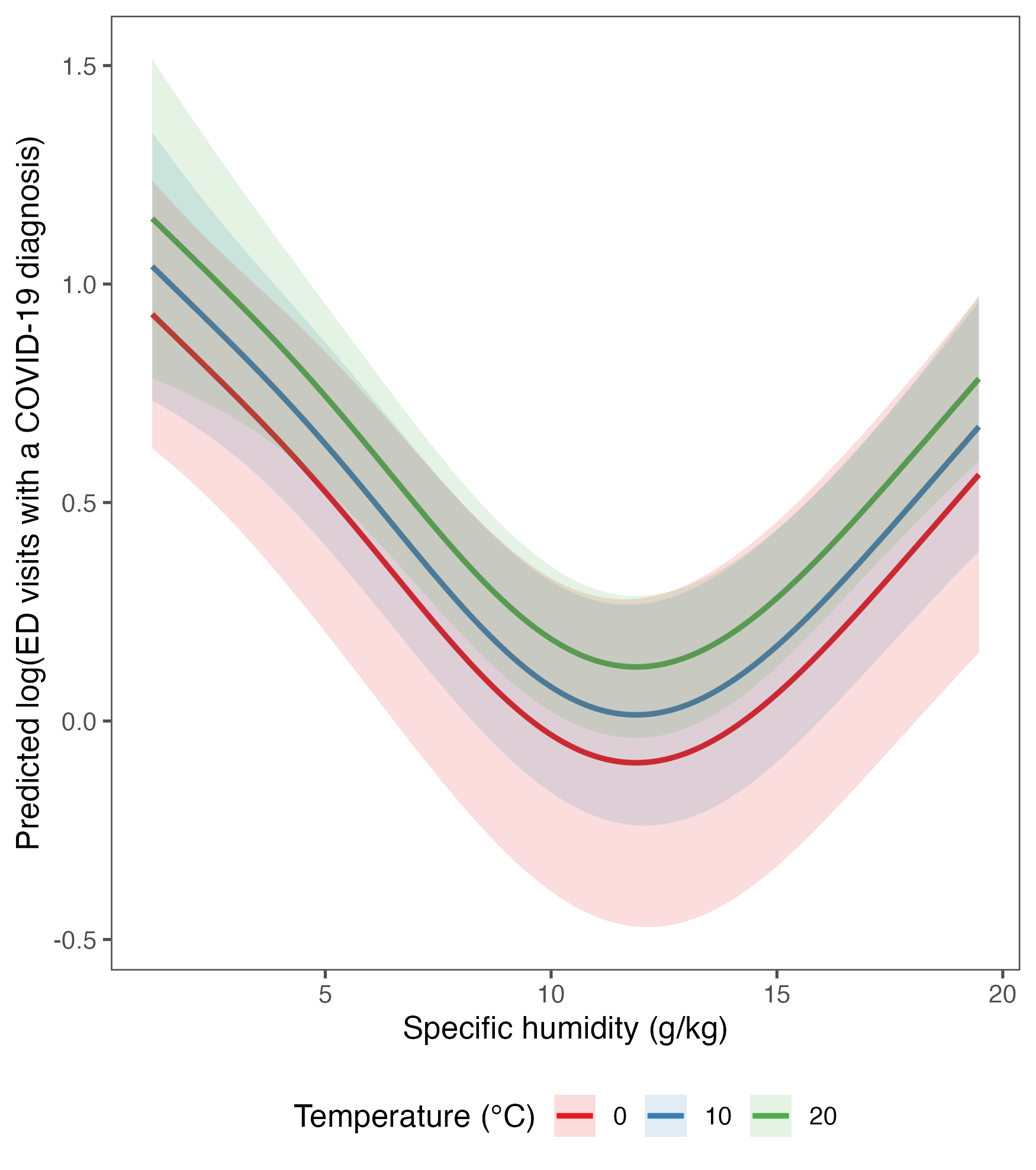

Supplement: S8 Fig — Estimated marginal effects at temperatures of 0 °C, 10 °C, and 20 °C. (PNG) [file pone.0342510.s008.png]
