# Supplementary material for: Spatial patterns and environmental influences of COVID-19 outbreaks, post-Omicron
Source: PLoS One. 2026 Feb 10;21(2):e0342510. doi: 10.1371/journal.pone.0342510 (PMC12890149; doi:10.1371/journal.pone.0342510)
Supplement: Attachment — Submitted filename: PLOS ONE COVID Response to Reviewers.docx [file pone.0342510.s014.docx]

We are thankful for the reviewers for raising some additional statistical and sensitivity analyses to strengthen the robustness of the findings. In response, we conducted a suite of robustness checks, including examining alternative outcome definitions and model specifications, and addressing potential sources of temporal and spatial bias. These analyses confirmed the stability of our primary results. We added four supplemental tables and three supplemental figures summarizing sensitivity analyses, model assumption checks, and annual state-level outbreak metrics. We believe these measures sketch out a more robust and comprehensive analysis investigating the association between climate factors and COVID-19 activity following the Omicron wave. Detailed, point-by-point responses are provided below.

Reviewer #1: The authors investigated the impact of climate patterns on COVID-19 outbreaks during the post-Omicron wave. The methods are appropriate, and the content is innovative. However, one point requires correction: the citation in Line 5 currently appears as “[?]” and should be fixed.

Thank you for noting this citation did not transfer, we have corrected it to link to the WHO COVID-19 dashboard, and is listed first in the references as: “COVID-19 deaths | WHO COVID-19 dashboard;. Available from: https://data.who.int/dashboards/covid19/cases.”

Reviewer #2: The manuscript analyzes U.S. state–level emergency department (ED) data (April 2022–March 2025) to describe post-Omicron COVID-19 seasonality and investigate associations with temperature and specific humidity using descriptive metrics (epidemic intensity and circular mean timing) and panel regressions/GAMs. It reports (i) a transparent latitudinal gradient in outbreak patterns and (ii) a U-shaped relationship between specific humidity and burden, with higher risk at both low and high values and lowest risk at intermediate humidity; temperature shows a more negative/monotone association overall. The analysis relies on public ED visit percentages from the CDC, ERA5 climate data, and state-level booster coverage, with figures summarizing temporal patterns and model-based response surfaces.

The topic is timely and suitable for PLOS ONE, and the dataset window (post-Omicron) is a strength. The manuscript offers a valuable post-Omicron perspective and clearly documents the geographic structure in COVID-19 ED patterns. However, several methodological decisions limit interpretability. With stronger attention to outcome denominators, serial/spatial dependence, bimodal timing, and potential confounders, in addition to fuller model transparency and robustness checks, the conclusions can be considered well-supported, and the claims about climate associations will be much more compelling and publication-ready.

Major comments

A. Using the percentage of ED visits with a COVID-19 diagnosis is defensible for mitigating testing/reporting artifacts. However, it is sensitive to changes in the ED case mix (e.g., heat waves, injuries, RSV/flu surges) that alter the denominator independent of COVID-19 transmission. Please:

- Re-run key analyses with an incidence-style outcome (e.g., estimated COVID-related ED visits per 100k population, if derivable from the CDC series) and/or with the log ratio of COVID ED visits to a stable internal control (e.g., total respiratory visits).
- At a minimum, control for state-week total ED volume (or include week×region fixed effects) to absorb denominator shifts. This control is crucial for interpreting the U-shape at high humidity/temperature in southern states, where summertime non-COVID ED volume is large.

Thank you for raising this important concern. To address potential denominator instability in the percentage of ED visits with a COVID-19 diagnosis, we re-estimated our primary GAM using an alternative outcome: the log ratio of weekly COVID-19 ED visits to weekly state-level ILI visits (CDC ILINet). ILI serves as a stable respiratory control that is much less likely to be affected by fluctuations in non-respiratory ED volume. We added one to ILI counts to accommodate zero-activity weeks. The revised GAM, identical to Equation 4 except for the outcome, produced highly consistent smooth terms for specific humidity and week (NEW Supplemental Table 1). The alternative model explained slightly more deviance (44.4% vs. 37.1%). These results indicate that our findings are robust to changes in denominator behavior.

(NEW) Supplemental Table 1. Comparison of climate smooth terms across outcome definitions: percentage of COVID ED visits (main model) vs. log ratio of COVID ED to ILI ED (robustness model).

B. EI is computed based on the mean weekly curve aggregated across April 2022 and March 2025. This collapses interannual variation (including variant waves) and may inflate smoothness. Please compute EI per COVID-year (Apr–Mar) by state, analyze variance across years, and show that the latitude relationship persists year by year. Sensitivity of results to this design choice should be presented (main text or Supplement).

This is an important point, as our original EI metric aggregated data across all COVID-years. In response, we recalculated EI separately for each COVID-year (Apr–Mar) and presented these results in NEW Supplemental Figure 2. Year-specific EI–latitude associations were generally consistent with the pooled estimates (Figure 1). The only exception was 2023-24, when strong winter peaks in northern states reduced the strength and significance of the association (p = 0.085). In both 2022-23 and 2024-25, latitude remained a strong, significant predictor (p <<< 0.001). These analyses demonstrate that the primary EI–latitude relationship is not an artifact of multi-year averaging.

(NEW) Supplemental Figure 2. Epidemic intensity calculated for each state and COVID-year in relation to latitude.

C. The paper summarizes timing with a single circular mean across years, but the distribution is explicitly biannual (summer and winter peaks). A single mean is not a sufficient statistic and can be misleading (means can land in troughs). Please report additional timing metrics: (a) circular dispersion, (b) separate peak weeks (summer/winter) per state/year, and (c) peak asymmetry (summer minus winter magnitude). Consider re-running the latitude regressions on these peak-specific outcomes.

We agree that the biannual structure of COVID-19 activity complicates interpretation of the mean case timing, which is typically most informative for pathogens with a single annual peak. Even so, we believe the mean case timing remains useful for indicating which peak - summer or winter - contributes more heavily to the annual distribution. States with more pronounced summer peaks tend to exhibit lower mean timing values, reflecting a greater concentration of cases during summer months. To contextualize this metric, we added an analysis of the winter-to-summer peak magnitude ratio for each COVID-year (NEW Supplemental Figure 2). Higher-latitude states exhibited larger winter-to-summer ratios, illustrating how relative peak strength varies geographically and across years. Together, these peak-ratio results complement and contextualize the mean case timing measure by illustrating how the relative strength of the two peaks shifts over time and across states.

(NEW) Supplemental Figure 3. Summer/Winter peak ratio for each state and COVID-year, where larger ratios indicate larger winter peaks relative to summer peaks.

D. Equation (3) includes state, year, and week fixed effects—a good start—but more detail is needed. Please:

- Clarify whether two-way or three-way FE are included simultaneously (state FE + year FE + week-of-year FE). If week FE is common across years, it absorbs seasonality; then the climate effect is identified through deviations from the seasonal mean. Make this explicit and discuss implications.
- Justify bin choices (5°C, 3 g/kg) analytically (e.g., using information criteria or cross-validation) rather than aligning reference bins to visual troughs (risk of post-hoc selection).
- Show partial dependence plots with CIs and within-R² to emphasize within-state identification.

Thank you for the opportunity to clarify the fixed effects models. Yes, fixed effects are included simultaneously such that the effects of temperature and specific humidity are identified through deviations from the seasonal mean. This is now clarified in the text.

To assess whether results were sensitive to binning decisions, we repeated the fixed-effects analyses across nine alternative binning strategies (temperature bins of 2°C, 3°C, 4°C, and 5°C; specific humidity bins of 2, 3, and 4 g/kg). Across all specifications, AIC, BIC, and cross-validated RMSE varied by less than 4%, and the estimated effects of temperature and specific humidity remained directionally and substantively unchanged (NEW Supplemental Table **2**). These results indicate that our chosen 5°C and 2 g/kg bins provide an appropriate balance of interpretability and statistical stability. Temperature followed an approximately normal distribution, while specific humidity exhibited a slight right-hand skew.

(NEW) Supplemental table 2: Sensitivity analyses to determine optimal temperature and specific humidity bin sizes.

Notably, we updated Figure 3a in the manuscript to include the updated bin sizes for specific humidity.

(UPDATED) Figure 3. Results from a binned fixed effects model assessing logged ED visits with a COVID-19 diagnosis. A. Results from the binned fixed effects model, showing a U-shaped relationship between specific humidity and logged ED visits, and a more linear relationship between temperature and logged ED visits. B. Surface plot indicating periods of predicted heightened COVID-19 activity from binned fixed effects model, where state-specific intercepts account for state-specific factors such as population density.

We included an updated partial dependence plot from the GAM to explore the marginal estimated effect of specific humidity on logged ED visits with at COVID-19 diagnosis, which is updated Supplemental Figure 3a (plot included in comment E response).

E. For the GAM [Equation (4)], please specify the exact basis, knot placement, smoothing parameter selection (REML is noted), and present edf, χ²/F-tests, and residual diagnostics (ACF of residuals, concurvity).

We appreciate the opportunity to elaborate and have now updated the GAM description to include more information. In the revised manuscript, we expanded the GAM description to specify spline bases, knot placement, smoothing parameter estimation (REML), and all inferential statistics. To address temporal autocorrelation, we added a cyclic cubic spline for epidemiologic week (s(week, bs=“cc”, k=20)), which enforces continuity across years. State fixed effects were included via factor(state). We now report edf, F-statistics, p-values, and concurvity metrics in NEW Supplemental Table 3 and provide full diagnostic plots in NEW Supplemental Figure 4. Diagnostics indicate approximate normality, minimal autocorrelation, and no major violations of model assumptions. We also added partial-effect plots for humidity and week (UPDATED Supplemental Figure 5).

(NEW) SI Table 3. GAM fit statistics.

Residual diagnostic plots, including residual vs. fitted, Q-Q plots, and the residual distribution, are included in (NEW) Supplemental Figure 4, confirming no remaining strong autocorrelation or systematic deviations from model assumptions. A Q-Q plot of deviance residuals (Supplemental Figure 4A) showed approximate normality with only minor tail deviations, indicating no major violations of distributional assumptions. Residuals had a normal distribution (Supplemental Figure 4B), and when plotted against the linear predictor (Supplemental Figure 4C) showed no systematic pattern or heteroskedasticity, indicating adequate mean-variance fit of the GAM. The response-fitted plot showed a positive linear association without systematic curvature (Supplemental Figure 4D), though with some residual variability, indicating that while the GAM captures broad mean patterns, climate factors alone explain only a modest share of week-to-week variation in ED visits with a COVID-19 diagnosis.

(NEW) Supplemental Figure 4. SI Diagnostic plots for the generalized additive model (Equation 4). A. Q-Q plot showing residuals largely following the theoretical normal distribution. B. Histogram of residuals, approximately symmetric and centered near zero. C. Residuals versus linear predictor, showing no major nonlinear patterns, with mild heteroskedasticity. D. Observed versus fitted values, indicating that fitted values span a narrower range than the observed log(ED visits). Together, these diagnostics support the adequacy of the model while motivating inclusion of state and cyclic week effects.

To interpret the shape and influence of the humidity spline, we present partial‐effect plots for the specific humidity and week-of-the-year spline terms for the generalized additive model (UPDATED Supplemental Figure 5).

(UPDATED) Supplemental Figure 5. Estimated smooth terms from the GAM including state and cyclic week effects. A. Specific humidity shows a nonlinear, U-shaped association with log(ED visits). B. The cyclic spline for week captures within-year seasonal structure. Solid lines represent fitted effects; dashed lines show 95% confidence intervals.

F. Weekly panel data will exhibit strong serial correlation. Clustering by state is necessary but not sufficient if residuals are highly persistent. Please report tests/plots of residual autocorrelation and repeat regressions with Driscoll–Kraay or Newey–West (panel) robust errors (or at least state-level AR(1) correction) to verify inference stability.

Thank you for bringing this to our attention, and we agree that weekly panel data can exhibit serial correlation. To address this, we re-estimated our model using Newey-West heteroskedasticity and autocorrelation (HAC) standard errors. We conducted a joint Wald test of the spline basis coefficients for both s(week_q) and s(week), and in both cases, the smooth terms remained statistically significant after the HAC correction, indicating that our primary inference remained robust to serial correlation. We report these HAC-adjusted tests with the GAM fit statistics in (NEW) Supplemental Table 3. Importantly, the initial conclusions of the GAM, being the strong nonlinear association between specific humidity and a nonsignificant linear effect of temperature, remain consistent under the HAC-robust analysis.

(NEW) SI Table 3. GAM fit statistics.

G. Neighboring states share weather and mobility catchments; ignoring spatial autocorrelation can bias SEs and inflate significance. Perform Moran’s I on residuals and, if material, implement spatial HAC corrections or include region×week FE. Alternatively, estimate a spatial error model on state-level annual summaries as a sensitivity analysis.

We appreciate the opportunity to look into potential spatial autocorrelation. We evaluated spatial autocorrelation in the state-level mean residuals from the GAM using global Moran’s I with border-or-corner adjacency (queen contiguity) weights (with 999 permutations). Moran’s I was -0.137 with a permutation p-value of 0.874, indicating no evidence of spatial clustering. This suggests that residual spatial dependence is minimal and does not substantially affect the findings. These results, combined with the inclusion of both state and week fixed effects and the Newey-West robust standard errors, indicate that our findings are stable to serial and spatial correlation.

H. Summer/winter peaks in ED COVID percentages may be entangled with RSV/flu/enterovirus waves, school terms, AC/indoor time, wildfire smoke, or holiday periods. At a minimum, add controls for:

- Influenza and RSV ED indicators (or ILI/SARI proxies) by state-week.
- Mobility or policy stringency (the Discussion notes this as future work; it belongs in robustness today).
- School in/out of session indicators.
- Wildfire smoke (PM2.5) has affected recent summers, particularly those with smoke-affected conditions. Include these in sensitivity models to demonstrate that climate associations are not proxies for unmeasured seasonal behaviors.

We thank the reviewer for raising this important point. We fully agree that COVID-19 patterns can be influenced by co-circulating respiratory pathogens, school schedules, holiday-related behavior, mobility, and other factors such as wildfire smoke. In fact, our week-level fixed effects control for average seasonal behaviors such as school semesters. Our robustness checks now include ED visits/ILI in response to prior suggestions. Post 2022 there were no nonpharmaceutical interventions implemented in the United States (CDC mask mandate for travelers no longer in effect following judge’s ruling, official says | CNN Politics. Accessed December 11, 2025. <https://www.cnn.com/2022/04/18/politics/cdc-mask-mandate-ruling/index.html>; CDC. CDC Museum COVID-19 Timeline. Centers for Disease Control and Prevention. July 8, 2024. Accessed December 16, 2025. <https://www.cdc.gov/museum/timeline/covid19.html>), however, our model does account for vaccination which was the main disease control tool implemented during this period. Although important, we believe that incorporating wildfire smoke data is beyond the intended scope of the manuscript and likely deserves a more focused analysis. However, we note that dropping major wildfire events from our dataset, specifically California in early 2025, PNW in 2025 and Canadian fires of 2023 does not affect our estimates (full model adjusted R^2^ value is 0.6136 including months with a wildfire vs. 0.6126 when excluding wildfire months in binned models).

I. The booster covariate is treated as a single percentage; please specify the series (which booster definition? updated 2023/2024 formulations?), data cadence, and lag structure. Consider lagged vaccination and age-adjusted coverage, as state averages can mask significant age/uptake differences. Show that results are robust to alternative vaccination specifications and to inclusion of prior-wave burden as a proxy for immunity.

Thank you for raising this important point regarding vaccination measurement. We obtained state-level COVID-19 vaccination and booster coverage from the CDC COVID-19 Vaccination Trends data catalog, which was last updated in May 2023. Because updates ceased at that time, we used the most recent available booster coverage value for each state-year (May 2022 for the 2022-23 season and May 2023 for the 2023-24 and 2024-25 seasons). These values represent the share of each state’s population with any completed booster dose as defined by CDC at the time of reporting; CDC does not provide historical series distinguishing updated formulations (e.g., 2023/24 XBB boosters). Because this vaccination adjustment served as a supplementary robustness check rather than a primary analysis, and given the absence of state-week age-specific or lagged booster data, evaluating lagged or age-adjusted vaccination coverage falls beyond the scope of the current study.

J. The paper occasionally reads as if climate “drives” the dynamics of COVID-19. Given the observational design, please temper language to “is associated with” throughout the Abstract, Author Summary, Results, and Conclusion, and emphasize that climate may “modulate” transmission conditional on variant immune escape and behavior. The Discussion begins to do this, but still infers “amplify” in places. Tighten wording, please.

We appreciate the reviewer’s suggestion to tighten the language around the use of the term “drivers.” In response, we carefully reviewed the manuscript and adjusted terminology to ensure that “drivers” is used only in contexts where it is scientifically warranted. Specifically, we now reserve the term for discussions grounded in mechanistic or laboratory evidence, references to established causal pathways, or instances where prior modeling work explicitly identifies climatic variables as transmission determinants. In all other contexts, particularly when interpreting correlational findings, describing ecological patterns, or summarizing associations, we adopted more neutral phrasing such as “influences,” “relationships,” or “associations.” We also revised language to distinguish between effects on epidemic intensity (a measure of outbreak concentration) versus incidence, to avoid overstating causal interpretation. Finally, to reflect this more precise language throughout the manuscript, we updated the title to “Spatial patterns and environmental influences of COVID-19 outbreaks, post-Omicron,” replacing the earlier use of “drivers” with “influences.” Together, these changes strengthen the clarity and scientific accuracy of the manuscript.

K. Since Apr 2022 covers multiple Omicron sublineages with differing immune escape, please include period or variant-era indicators (or break the sample) and test for parameter stability (e.g., climate coefficients pre- vs post-XBB/BA.5 transitions). A figure that locates major lineage turnovers on the time axis would be helpful.

Thank you for this thoughtful suggestion. We agree that SARS-CoV-2 variant turnover could influence the magnitude and timing of outbreaks, and we intentionally began our analytic window in April 2022 to focus on the period following the major Omicron BA.1/BA.2 wave. Our goal was to characterize emerging seasonal patterns after the large-scale shifts in population immunity associated with the initial Omicron transition, rather than to model variant-specific dynamics. The study period therefore spans multiple Omicron sublineages (including BA.5, BQ.1, XBB lineages, and JN.1) with differing immune-escape properties. While these transitions are important epidemiologically, incorporating variant-era indicators or estimating pre-/post-transition climate coefficients is beyond the scope of the current analysis, which aims to evaluate broad seasonal patterns rather than lineage-specific transmission mechanisms.

L. The fixed-effects bins suggest a U-shape for humidity and a negative slope for temperature; the GAM surface then shows the highest predicted burden at the high-humidity/high-temperature corner for Florida and at cold/dry for Wisconsin. To avoid over-interpretation:

- Quantify uncertainty on the surface [e.g., contours of standard error of the mean (SEM) or bootstrap].
- Show the empirical distribution of (T, q) combinations (you plot a polygon of support; add heat-density so readers see where the model extrapolates).
- Report marginal effects of humidity at several temperature strata (and vice versa).

Thank you for the opportunity to clarify. We include partial response plots for the GAM specific humidity and week-of-the-year spline terms (UPDATED Supplemental Figure 6). Adding contours to the figure using bootstrapped standard errors resulted in a visually busy plot that was difficult to interpret. To further contextualize the observed climate space represented in the data, we created NEW Supplemental Figure 2, which shows the joint distributions of temperature and specific humidity. To avoid over-complicating Figure 3b, we display only the outline of the observed distribution.

(NEW) Supplemental Figure 2. Joint distribution of observed temperature and specific humidity values across all states in dataset.

Finally, we visualize the marginal effect of specific humidity at temperatures of 0 °C, 10 °C, and 20 °C in NEW Supplemental Figure 8, illustrating how logged ED visits with a COVID-19 diagnosis are estimated to vary across temperature strata. We believe these additions improve interpretability while preserving clarity and appropriately convey both the modeled relationships and the underlying data support.

(NEW) Supplemental Figure 8. Marginal effect of specific humidity on the predicted logged ED visits with a COVID-19 diagnosis.

M. Given literature heterogeneity (absolute vs. relative humidity, dew point, WBGT, humidex, specific enthalpy, UV), include a sensitivity table swapping relative humidity, dew point, and a solar/UV proxy (ERA5 clear-sky UV if available) to show that findings are not metric-dependent.

We agree that multiple related climate variables (e.g., specific humidity, relative humidity, temperature, dewpoint temperature) may be associated with our outcome of COVID-19 activity, and that assessing robustness across metrics is important. To address this, we reestimated the GAM using alternative specifications using climate variables available in ERA5 data: relative humidity and dewpoint temperature. Across all model specifications, the estimated functional relationship remained stable, and the results were highly consistent with the base model that used specific humidity. Adjusted R^2^ values ranged from 0.34 to 0.37 across models, and the smooth terms for specific humidity remained statistically significant in all cases. These findings indicate that our results are not dependent on the specific humidity and temperature metrics chosen, and that our initial model specification accounted for the largest amount of variability in the observed data. We included (NEW) Supplemental Table 4 which included GAM fit statistics from the various model specifications.

(NEW) SI Table 4. Sensitivity of GAM results to alternative humidity and temperature specifications

Minor comments

Recommendations for figures:

- Fig 1: Note Wyoming data truncation explicitly in the caption.
- Fig 2: For heatmaps and grouped curves, include N(states) per subgroup and shaded uncertainty (e.g., state bootstraps).
- Fig 3: The caption has “Fig 3. Fig 3.” duplicated; fix. Overlay monthly points with error bars for Florida/Wisconsin and add a legend for the support polygon. Provide a color bar with units (log-percent ED COVID).

Thank you for this suggestion. We expanded the Methods section to clarify ERA5 aggregation, centroid calculations, and state exclusions. We now specify state-level averaging, centroid computation using st_centroid(), and exclusion of Alaska and Hawaii.

Recommendations for reproducibility & data availability:

- The GitHub link and statement are helpful; please archive a frozen release (e.g., Zenodo or similar) with a DOI, include environment lockfiles (R version noted as 4.4.3; also pin package versions), and provide download scripts for the ERA5 and CDC series so that others can exactly reproduce your extraction window and state mappings.
- Clearly document which CDC endpoints were used, their as-of date, any backfill handling, and whether data revisions occurred during analysis.

Thank you for this opportunity to clarify; we have updated our description of which CDC data were used in the analysis. We have also updated the GitHub code to reflect the analytic changes, including a new section of the sensitivity analyses.

“N/A” is written in the Ethics Statement section. Because the analysis utilizes publicly available, aggregate surveillance data, add a sentence in the Methods section stating that the data are de-identified and publicly available, and that no human subjects review was required (cite the data sources).

Thank you for this suggestion, we believe citing the source of the data is sufficient.

Ensure the funding statement appears verbatim in the manuscript Acknowledgments/Financial Disclosure as required by PLOS ONE.

Thank you for this comment, we confirmed the funding statements are the same in the Acknowledgments and Financial Disclosures sections.

#5: Replace the “1.2 million deaths as of September 2025 [?]” citation placeholder with a verifiable source.

Thank you for noticing this reference was invalid, we updated it to link to the WHO COVID-19 dashboard, and is listed first in the references as: “COVID-19 deaths | WHO COVID-19 dashboard;. Available from: https://data.who.int/dashboards/covid19/cases.”

Fix typos (e.g., “the the stringency index” in #191) and grammatical errors (e.g., “Data on COVID-19 vaccination data on the state level …” in #55 and #56 OR “becomes” in #128).

Thank you for picking up on these instances. We have extended the description of the stringency index, including linking it to the Oxford Covid-19 Government Response Tracker which is the organization that coined the index. We now provide more information about the Oxford Covid-19 Government Response Tracker, and provide more context to describe the stringency index. When describing COVID-19 data, we now say, “Data on COVID-19 vaccination information at the state level were downloaded from the CDC COVID-19 Vaccination Trends data catalog”. We have also updated language in line 128 as noted to improve the grammar.

In Materials and methods, clarify spatial aggregation: ERA5 0.25° grids → state-level averages (population-weighted or area-weighted?). Specify the state centroid source for latitude and whether Alaska/Hawaii were included or excluded.

We appreciate this opportunity to expand our description in the methods. We updated the text to detail the state centroid calculation and exclusion of Alaska and Hawaii. The text now reads: “ERA5 hourly reanalysis data for 2 m temperature and dew point temperature were downloaded at 0.25° × 0.25° resolution (roughly 30 km$^2$ grids), and daily values were extracted for each state using state-level shapefiles \cite{tigris}. Specific humidity and relative humidity were computed from temperature and dew point temperature using standard Magnus formulations for actual and saturation vapor pressure, which are approximations of the Clausius-Clapeyron relation. Centroids for each state (representing the geographic midpoint of their latitude and longitude coordinates) were calculated using the st_centroid() function from the *sf* package. Data on the weekly percentage of ED visits with a COVID-19 diagnosis at the state level were downloaded from the CDC COVID Data Tracker. Data on COVID-19 vaccination information at the state level were downloaded from the CDC COVID-19 Vaccination Trends data catalog. Alaska and Hawaii were excluded to focus analyses on contiguous US states. A complete-case analysis was conducted, excluding Wyoming in 2024-25 due to discontinuation of state-level reporting.”

Please note missingness: e.g., Wyoming stopped reporting in 2025; describe how weeks with missing ED data are handled (listwise deletion? imputation?). Provide a completeness table by state×year.

Thank you for this opportunity to clarify how we handled missing data from the CDC COVID Data Tracker. For this investigation, we conducted a complete-case analysis and excluded Wyoming in 2024-25 due to discontinuation of state-level reporting. Supplemental Figure 1 illustrates data completeness across states. To clarify concerns about missing ED data, we added a sentence in the data section of the methods, stating: “A complete-case analysis was conducted, excluding Wyoming in 2024-25 due to discontinuation of state-level reporting”.
